# Beyond right ventricular injury: toward a full-chain right-heart monitoring framework in acute respiratory distress syndrome

**DOI:** 10.1093/ajrccm/aamag322

**Published:** 2026-07-10

**Authors:** Yi Li, Wanhong Yin, Xiaoting Wang

**Affiliations:** Department of Critical Care Medicine, West China Hospital, Sichuan University, Chengdu, PR China; Visualized Diagnostics and Therapeutics & Artificial Intelligence Laboratory (VDT-AI Lab), West China Hospital, Sichuan University, Chengdu, PR China; Department of Critical Care Medicine, West China Hospital, Sichuan University, Chengdu, PR China; Visualized Diagnostics and Therapeutics & Artificial Intelligence Laboratory (VDT-AI Lab), West China Hospital, Sichuan University, Chengdu, PR China; Department of Critical Care Medicine, Peking Union Medical College and Chinese Academy of Medical Sciences, Beijing, PR China

To the Editor

We read with great interest the concise clinical review by Slobod and colleagues on right ventricular (RV) hemodynamics in acute respiratory distress syndrome (ARDS).^1^ Their article provides an important synthesis of multimodal RV monitoring and rightly emphasizes that RV injury in ARDS is dynamic, prognostically relevant, and potentially modifiable. We particularly appreciate the authors’ effort to move the bedside assessment of right heart injury in ARDS with high mortality rates^2^ beyond isolated echocardiographic description toward integrated physiologic monitoring.

We would further argue that RV assessment in ARDS should be framed as a full-chain right-heart monitoring strategy organized around the physiologic “front-back-left-right” determinants of the right ventricle rather than around RV dysfunction alone. The right ventricle is influenced anteriorly by venous return (“front”), posteriorly by the pulmonary circulation and afterload (“back”),^3^ leftward by ventricular interdependence (“left”), and rightward by its own chamber structure and mechanics (“right”).^4^ In this schema, the “back” is often the cause in ARDS because pulmonary vascular loading, lung mechanics, recruitability, hypoxemia, hypercapnia, and pulmonary vascular obstruction are frequently the proximal drivers of RV injury. By contrast, reduced venous return, systemic congestion, RV dilatation, septal shift, or a low tricuspid annular plane systolic excursion (TAPSE) are often downstream manifestations rather than the primary lesion.^5^ For this reason, RV dysfunction should not be evaluated in isolation; it should trigger comprehensive monitoring across the full chain.

This distinction is clinically important because afterload-driven RV injury can easily be misread as a problem of contractility or preload alone.^3^^,^^4^ A single RV-focused snapshot remains incomplete if it is not interpreted together with plateau pressure, driving pressure, positive end-expiratory pressure (PEEP)–related overdistension or derecruitment, gas exchange, pulmonary vascular pathology, and the degree of RV–left ventricular (LV) interaction.^3^ In practice, right over left ventricular end-diastolic area (RVEDA/LVEDA) ratio, septal flattening, TAPSE, or fractional area change should be read together with respiratory mechanics, blood gases, prone response, and the hemodynamic consequences of ventilator adjustment.

We also suggest that acute cor pulmonale should be treated less as a static echocardiographic label and more as an actionable hemodynamic phenotype. The bedside question is not only whether acute cor pulmonale is present, but whether it is producing forward failure, backward failure, or both.^5^ Forward failure can be tracked by stroke volume surrogates and systemic perfusion. Backward failure requires explicit evaluation of right atrial pressure transmission and organ congestion, ideally using hepatic, portal, and renal venous Doppler or a venous excess ultrasound (VExUS)–based approach.^6^ Such a full-chain framework may help avoid iatrogenic fluid loading in patients whose dominant problem is RV limitation and venous congestion rather than preload deficiency. The proposed full-chain framework and acute cor pulmonale thinking guidance are summarized in Figure 1.

**Figure 1:**
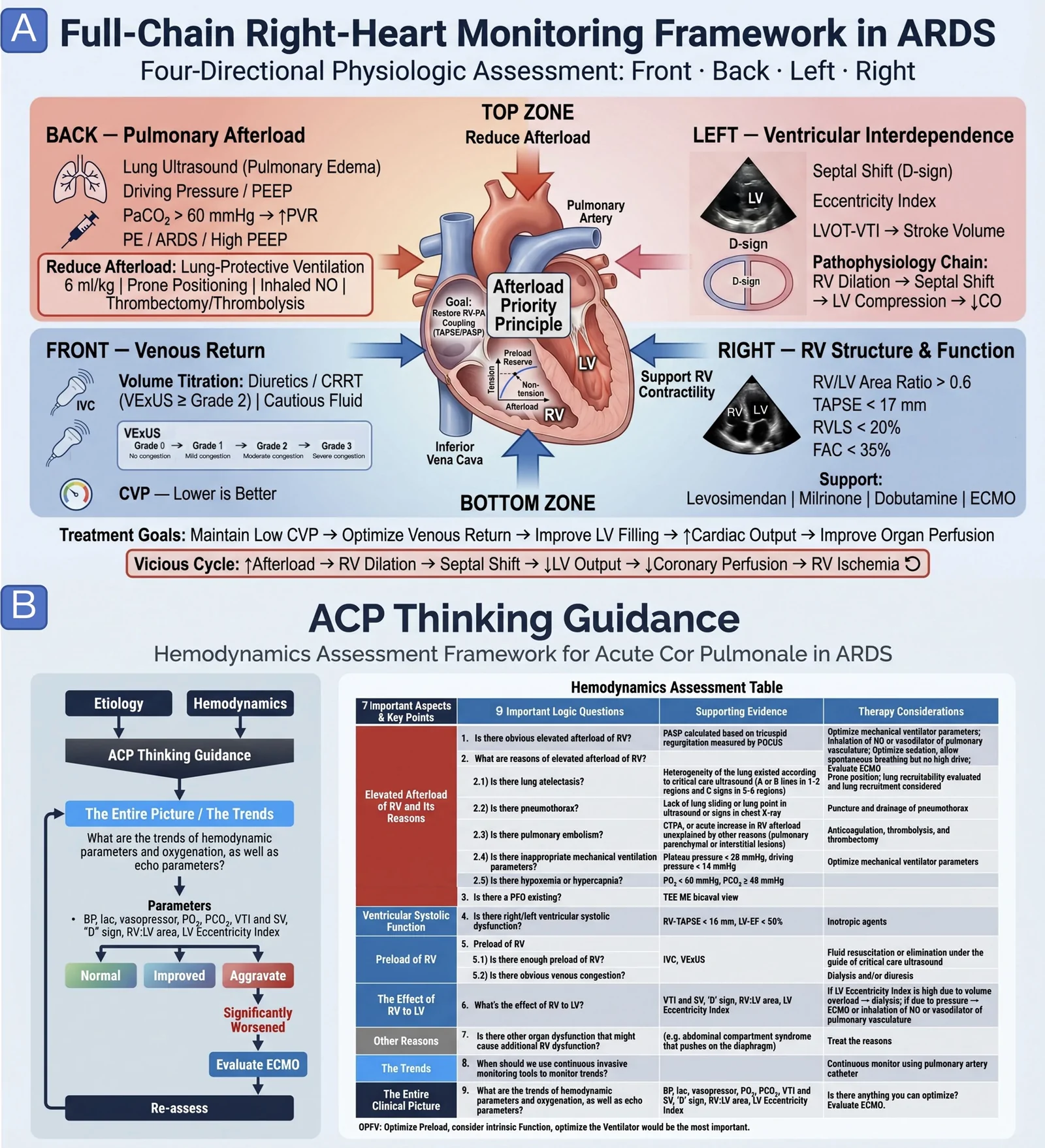
Schematic illustration of a bedside full-chain right-heart monitoring strategy in ARDS. Assessment is organized into 4 physiologic domains—front, back, left, and right—linking venous return, pulmonary afterload, ventricular interdependence, and RV structure/function. The figure highlights that afterload is the dominant upstream determinant of RV injury in ARDS and that ACP thinking guidance may help translate multimodal monitoring into targeted, iterative management. (A) Full-chain right-heart monitoring framework in ARDS. (B) ACP thinking guidance for bedside hemodynamic assessment in ARDS. Abbreviations: ACP, acute cor pulmonale; ARDS, acute respiratory distress syndrome; BP, blood pressure; CO, cardiac output; CRRT, continuous renal replacement therapy; CTPA, computed tomography pulmonary angiography; CVP, central venous pressure; ECMO, extracorporeal membrane oxygenation; FAC, fractional area change; lac, lactate; LVOT-VTI, left ventricular outflow tract velocity-time integral; LV, left ventricular; NO, nitric oxide; PASP, pulmonary artery systolic pressure; PE, pulmonary embolism; PEEP, positive end-expiratory pressure; PFO, patent foramen ovale; PVR, pulmonary vascular resistance; RV, right ventricular; RVLS, right ventricular longitudinal strain; SV, stroke volume; TAPSE, tricuspid annular plane systolic excursion; TEE ME, transesophageal echocardiography mid-esophageal view; VExUS, venous excess ultrasound; VTI, velocity-time integral.

Accordingly, we propose a pragmatic 4-step bedside approach in ARDS: identify the dominant source of RV afterload elevation; define the RV phenotype and the degree of RV–LV interaction; quantify systemic venous congestion and its organ consequences; and reassess the entire chain after interventions such as prone positioning, individualized PEEP adjustment, inhaled pulmonary vasodilators, or fluid removal. In our view, this extension is aligned with the authors’ review and may help translate multimodal monitoring into more precise and less RV-injurious management.

## Supplementary Material

aamag322_Supplementary_Data

## Data Availability

No new data were generated or analyzed for this correspondence.
